# Comparing Conventional and Advanced Approaches for Heavy Metal Removal in Wastewater Treatment: An In-Depth Review Emphasizing Filter-Based Strategies

**DOI:** 10.3390/polym16141959

**Published:** 2024-07-09

**Authors:** Jana Ayach, Wassim El Malti, Luminita Duma, Jacques Lalevée, Mohamad Al Ajami, Hussein Hamad, Akram Hijazi

**Affiliations:** 1 Research Platform for Environmental Science (PRASE), Doctoral School of Science and Technology, Lebanese University, Beirut P.O. Box 6573/14, Lebanon; jana.ayach.1@st.ul.edu.lb (J.A.); mohamad.r.alajami@hotmail.com (M.A.A.); akram.hijazi@ul.edu.lb (A.H.); 2 CNRS, ICMR UMR 7312, Université de Reims Champagne-Ardenne, 51687 Reims, France; luminita.duma@univ-reims.fr; 3 College of Health Sciences, American University of the Middle East, Egaila 54200, Kuwait; 4 CNRS, IS2M, UMR 7361, Université de Haute-Alsace, 68100 Mulhouse, France

**Keywords:** clean water, filters, heavy metals, SDG6, wastewater

## Abstract

Various industrial activities release heavy metal ions into the environment, which represent one of the major toxic pollutants owing to their severe effects on the environment, humans, and all living species. Despite several technological advances and breakthroughs, wastewater treatment remains a critical global issue. Traditional techniques are dedicated to extracting heavy metal ions from diverse wastewater origins, encompassing coagulation/flocculation, precipitation, flotation, and ion exchange. Their cost, side toxicity, or ineffectiveness often limit their large-scale use. Due to their adaptable design, simple operation, and reasonable cost, membrane filtration and adsorption have proven their efficiency in removing metals from wastewater. Recently, adsorption-based filters have appeared promising in treating water. Within this range, filters incorporating natural, synthetic, or hybrid adsorbents present an appealing alternative to conventional approaches. This review aims to list and describe the conventional and advanced wastewater treatment methods by comparing their efficiency, cost, and environmental impact. Adsorption-based filters were highlighted due to the significant advantages they can provide.

## 1. Introduction

Over time, environmental pollution has witnessed a persistent rise, impacting both human existence and the broader ecosystem. Water pollution is growing mainly due to wastewater from various industries dealing with paper and pulp, textiles, chemicals, etc. [1]. As of July 2022, a report jointly published by the World Health Organization and the United Nations International Children’s Emergency Fund (UNICEF) revealed that approximately 2.2 billion individuals remain without access to clean and safe drinking water [2]. Additionally, around 653 million people lack hand-washing facilities in their homes. The makeup of pollutants found in water effluents is contingent upon the industries in the vicinity responsible for generating the wastewater [3]. These effluents often contain harmful substances such as pesticides, dyes, aromatic hydrocarbons, oils, heavy metals, and more, which, when released into the environment, pose detrimental consequences for both humans and wildlife [4,5,6]. In addition, the continuous industrialization in developing countries significantly increased water demand [7]. Among several alternatives to preserve water resources, developing efficient and sustainable wastewater treatment technologies can provide a convenient way to reuse it [1].

Efficient wastewater treatment, encompassing the removal of heavy metals and their responsible utilization, plays a vital role in attaining the United Nations Sustainable Development Goal 6 (SDG6) of universal access to drinking water, sanitation, and hygiene by 2030 [8]. Wastewater treatment practices vary from one nation to another, exhibiting significant distinctions, particularly between developed and developing countries. While advanced countries mainly use centralized treatment, many developing countries use decentralized or no wastewater treatment. Although centralized treatment has some advantages, it may not be sustainable in the long term because of its high environmental impact related to resource consumption. In contrast, built-up treatment plants can be a sustainable solution for all countries, regardless of their development level. Most importantly, constructed washes may be a sustainable wastewater treatment for developed and emerging countries [9].

The pollutants generated in the water effluents are usually classified as organic and inorganic contaminants, depending on their nature and toxicity levels. Heavy metals are highly toxic inorganic pollutants, non-biodegradable, and their accumulation in wastewater yields a weighty environmental burden [10]. These heavy metals come from multiple industrial activities such as tannery and battery manufacturers, electroplating, mining, pesticides, and paint industries [11]. Hence, they stand out as the most enduring contaminants within wastewater, and their elevated toxicity can potentially impact critical bodily functions, including blood composition, brain activity, respiratory system, kidneys, liver, and other vital organs [12]. As a result, extended exposure may lead to the gradual onset of physical, muscular, and neurological degenerative conditions that exhibit similarities to diseases such as Alzheimer’s, Parkinson’s, muscular dystrophy, and multiple sclerosis [9,10]. As a result, heavy metals significantly harm human health, aquatic ecosystems, and the surrounding environment. Thus, it is becoming crucial to simultaneously reduce these numerous pollution sources, develop effective techniques for eliminating such dangerous pollutants from wastewater, and recycle them for other uses.

Several conventional methods, including coagulation, osmosis, ion exchange, and precipitation, are employed to decrease the elevated concentration of various metal ions in wastewater to meet the required regulatory levels [13]. However, many factors often limit these methods, such as cost and toxic environmental side effects [14]. Other relatively advanced techniques, such as adsorption and membrane filtration, have become excellent alternative treatments with improved removal effectiveness and reduced costs and environmental impact [15]. Indeed, the effective removal of heavy metal ions from wastewater necessitates the consideration of several critical factors, including their concentration, specific metal types, pH levels, chemical additives, removal efficiency, environmental consequences, and cost implications. In industrial settings, adsorption is widely acknowledged as one of the most efficient methods for wastewater treatment, effectively reducing the presence of harmful inorganic and organic pollutants in the treated wastewater released as effluents [16]. Heavy metal ions’ adsorption results from physicochemical interactions, primarily involving ion exchange or the formation of complexes between the metal ions and the functional groups found on the surface of the adsorbent. Furthermore, cost-effective adsorbents derived from natural biomass materials, industrial solid waste, agricultural byproducts, and biosorbents as source materials emerge as promising alternatives [16,17]. They exhibit enhanced adsorption capabilities after subjecting raw biomass adsorbents to various physical or chemical treatments [18,19,20]. Nevertheless, natural, synthetic, and hybrid filters (a mix of natural and synthetic) show high adsorption capacity as they efficiently adsorb heavy metals and dye pollutants in aqueous solutions.

In this review, we have compiled and compared examples of the most common conventional and relatively advanced wastewater treatment methods used to remove heavy metals. We have described the most relevant features of the removal methods and discussed their performance with respect to the cost, environmental impact, and removal efficiency. In addition, we have focused on advanced techniques, especially membrane filtration and adsorption, and highlighted the advantages of the different filter types and applications.

## 2. Heavy Metals: Sources and Toxicity

Heavy metals in surface water systems can have natural origins or result from human activities. Geological or natural sources encompass volcanic eruptions, the weathering of rocks containing metals, sea salt spray, wildfires, and natural weathering processes that can initiate the release of metals into different environments. Heavy metals can take various forms, including hydroxides, oxides, sulfides, sulfates, phosphates, silicates, and organic compounds [21,22]. Furthermore, various industrial manufacturing processes discharge heavy metals, including Cr, Cd, Ni, Cu, Zn, As, and Pb, at varying toxicity levels. These metals exhibit high water solubility and can be absorbed by aquatic organisms, leading to their accumulation in the human body via the food chain. Exceeding the required concentrations of heavy metals when ingested can result in various health disorders [23]. Heavy metal contamination poses environmental risks due to its carcinogenic, teratogenic, and endocrine-disrupting effects, which can significantly impact children’s behavior [24].

Table 1 summarizes the major sources and effects of common heavy metals, excluding cadmium, copper, and lead. The latter particular metals are discussed in detail in the following due to their prevalence in water contamination and the significant attention they have received over the decades.

Cadmium (Cd), a profoundly toxic metal, is primarily produced as a byproduct of zinc manufacturing. It becomes stored in plants and subsequently ingested by microorganisms and humans. Exceeding the threshold level (7 µg/L) of cadmium can lead to harmful effects. The metal shows a high transfer rate from soil to plants, resulting in elevated cadmium levels in fruits and vegetables. As a non-essential toxic metal, it significantly disrupts cellular enzymatic systems, leading to oxidative stress and nutrient deficiencies in plants. Additionally, cadmium is associated with hepatotoxicity, causing liver disease, by binding to cysteine-rich proteins like metallothionein. Furthermore, cadmium has an affinity for binding with ligands like glutamate, histamine, and aspartate, potentially causing iron deficiency [25].

Copper (Cu) exists in the environment due to natural and human activities. Anthropogenic sources of copper include mining, metal and electric production, fungicide use, leather processing, and vehicle brake pads. Also, natural copper contamination sources can arise from forest fires, volcanic eruptions, and windblown dust [26]. Copper is involved in many enzymatic systems in the organism but becomes toxic at high intake levels, exceeding 2000 µg/L [27]. Copper is crucial for nutrition and plays a vital role in brain function, but its presence is also associated with the development of Alzheimer’s disease. Even small concentrations of copper in drinking water can notably impact individuals’ learning and memory [28]. Moreover, prolonged exposure to copper primarily affects the liver, the first site where copper accumulates upon entering the bloodstream. Copper toxicity typically manifests as the development of cirrhosis, accompanied by episodes of hemolysis and damage to renal tubules, and affects the brain and other organs. Prolonged and excessive exposure to copper can lead to hepatic necrosis, vascular collapse, coma, and ultimately, fatality [29].

Lead (Pb) primarily enters the environment through various sources such as lead paint, house dust, food, air, soil, pottery, solder, porcelain, and tin. It accumulates in the body and can lead to disruptions in the number of red blood cells and kidney and brain diseases. It poses a significant hazard to young children and pregnant women, affecting physical development and normal mental growth in adults. Drinking water contaminated with lead (threshold of Pb: 25 µg/L) harms human health, especially for children consuming fruit juice and foods prepared with water containing lead [30,31,32].

**Table 1:** Common heavy metal ions in wastewater: sources and effects.

Heavy Metal	Major Source(s)	Common Effects	Reference
Arsenic (As)	Insecticides and pesticides, treated wooden pulps, and smelting metals.	Cancer, skin color changes, Blackfoot disease, diabetes, and others.	[31,32]
Zinc (Zn)	Plating, galvanizing, other metal finishing processes, paper and pulp industries, battery industry.	Kidney failure, lung fibrosis and cancer, anemia, vomiting, and others.	[33]
Chromium (Cr)	Electroplating, leather tanning, and textile industries.	Dermatitis, kidney and gastric damage, lung cancer, respiratory tract and eyes irritation, and others.	[34,35]
Mercury (Hg)	Oil refinery processes, pesticides, and burning coal for power generation.	Brain, central nervous system, heart, alimentary tract, kidney, and liver significant damages.	[36]
Nickel (Ni)	Electroplating, printing and dyeing, and pharmaceutical and metallurgical industries.	Skin irritation, asthma, conjunctivitis, and cancer.	[37,38]
Bismuth (Bi)	Chemical and pharmaceutical industries.	Hypotension, insanity, and renal failure.	[39]

## 3. Elimination of Heavy Metals from Wastewater: Conventional Methods

A variety of traditional methods are employed for the extraction of heavy metals from wastewater. These techniques include coagulation and flocculation, chemical precipitation, ion exchange, flotation, electrochemical separation, and evaporation [40]. In the following, we describe the most common and significant conventional practices for wastewater treatment, along with their advantages and disadvantages.

### 3.1. Coagulation and Flocculation

Coagulation refers to destabilizing colloids by neutralizing their repulsive forces, while flocculation involves aggregating unstable particles [41]. Traditional coagulants such as aluminum, ferrous sulfate, and ferric chloride are employed to counteract ionic charges. On the other hand, flocculation entails the amalgamation of particles to form larger coagulates. These larger coagulates can be generated using materials like polyaluminum chloride, polyferric sulfate, polyacrylamide, and various polymer flocculants [42]. The coagulation-flocculation process demonstrated both effective performance and straightforwardness in removing heavy metals from wastewater [43]. According to previous works, polyethylene-glycol is one of the most practical flocculants [44]. This approach can efficiently eliminate a range of heavy metals, including Cu, Pb, Ni, As, Se, Cr, Sb, and Ag [45]. The removal efficiency for the mentioned heavy metal ions varied between 95% and 99%. However, there are several drawbacks associated with the application of flocculation that can be pinpointed. These include the health hazards posed by inorganic coagulants, the generation of significant amounts of sludge, limited selectivity for specific metals and inefficiency in addressing emerging pollutants, the potential for increased sewage colors, lower effectiveness when employing natural coagulants, and challenges in scaling up the process [46].

### 3.2. Precipitation

**Figure 1:**
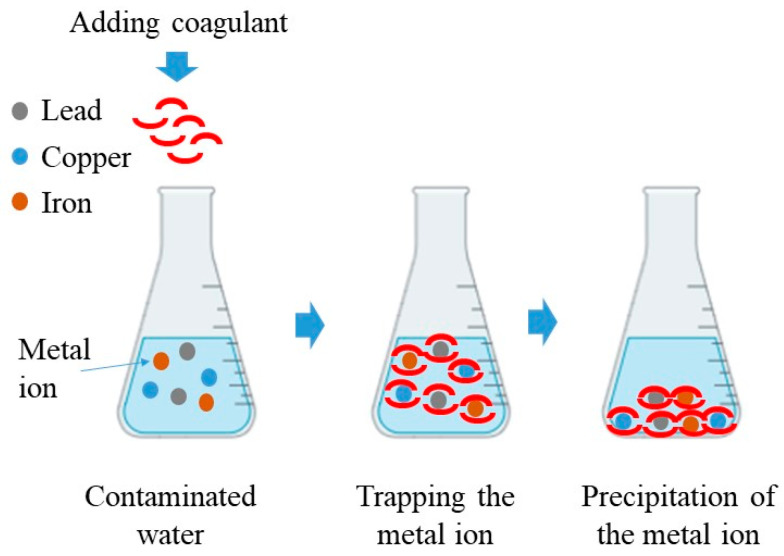
The precipitation technique for extracting heavy metals from wastewater.

The chemical precipitation method, also known as coagulation precipitation, is extensively utilized in various industries and is regarded as one of the most efficient and well-established techniques. This method involves transforming dissolved metal ions into solid particles, facilitating precipitation (Figure 1). The coagulant precipitates metal ions in many ways, such as changing pH, electro-oxidation potential, or co-precipitation, followed by removing deposits.

Hydroxide precipitation is commonly utilized due to its cost-effectiveness, simplicity, and the ability to adjust pH as needed [46]. This is achieved by adding hydroxide to the agitated wastewater, forming insoluble metal hydroxide precipitates. For example, metal ions can react with calcium hydroxide (lime), producing metal hydroxide precipitates and calcium ions, as illustrated in Equation (1).

(1) $$M^{n+}+{Ca(OH)}_{2}⇆{M(OH)}_{n(s)}+{Ca}^{2+}$$

In addition, sulfides are widely used to precipitate the metals polluting the wastewater (Equation (2)).

(2) $$M^{n+}+S^{2-}⇆M_{n}S_{\left(s\right)}$$

Despite its relatively high efficiency, about 95–99%, for most heavy metals present in wastewater, the precipitation method suffers various drawbacks [47]. High pH dependence and difficulty in removing large sludge volumes from the water are among the most critical limitations [48].

### 3.3. Ion Exchange

The reversible chemical response used to transform unwanted metallic ions into less environmentally harmful ones makes the ion exchange treatment an alternative to the previously described methods [49]. A heavy metallic ion is eliminated from the wastewater by bonding to a motionless solid particle as an alternative to the solid particle cation. The ion-alternate technique can put off-target (a few or all) heavy metallic ions, including Pb^2+^, Hg^2+^, Cd^2+^, Ni^2+^, V^4+^, V^5+^, Cr^3+^, Cr^4+^, Cu^2+^, and Zn^2+^ from wastewater. The ion exchange occurs when the alternate cation of the ion exchanger exchanges the wastewater pollutant cation, as shown in Equation (3), where M^−^EC^+^ represents the ion exchanger; EC^+^, the exchange cation; WC^+^, the wastewater cation; and M^˗^, the stable ion. Na^+^ and H^+^ are usually used as alternate cations [50].

(3) $$M^{-}{EC}^{+}+{WC}^{+}⇆M^{-}{WC}^{+}+{EC}^{+}$$

Various types of resins have been investigated for cation removal and described in the literature, such as Amberlite IRC86 chelating exchange resin [51] and Diaion weak cationic resin CR11 [52]. Both examined resins demonstrated an efficiency of approximately 90% in the removal of trivalent chromium from aqueous solutions [52]. In addition, zeolite exhibits an outstanding ion exchange capacity of up to 98% due to its inherent negative charge. This negative charge arises from Si^4+^ in the center of the tetrahedron, which undergoes isomorphous replacement with Al^3+^ cations. Furthermore, metal-organic frameworks (MOFs) have been extensively studied and proposed as highly promising materials for the ion exchange removal process [53]. Some reported MOFs were used for Cd^2+^, Pb^2+^, and Hg^2+^ removal with a capacity of 97%, 95%, and 93%, respectively [54], ZIF-8 eliminated 95% of Cu^2+^ [55], and ZIF-67 eliminated 98% of Cr^6+^ [56]. Although the ion exchange method has shown promising results, further research is needed to investigate its stability and reusability [44].

### 3.4. Flotation

Flotation is a gravity separation typically implemented to separate minerals, such as sulfides. This procedure holds substantial importance in the economies of all developed nations [57,58]. Nowadays, it is used extensively for metal ion recovery from industrial wastewater and is applied to a wide range of heavy metal waste streams [59,60]. Several types of flotation separation have been discussed in the literature, such as dissolved-air flotation and ion flotation, which are considered the most significant [61].

The dissolved air flotation consists of applying gas bubbles to the body of wastewater, which will selectively transfer the metal ions to the water’s surface, forming a slug that can be easily eliminated. This process helped remove heavy metals, such as Cu, Cd, Fe, Ni, Mo, Mn, and As, from wastewater with good efficiencies [61].

On the other hand, ion flotation was implemented to eliminate Cr^3+^, Pb^2+^, and Cu^2+^ from wastewater in the presence of different surfactants. Removal effectiveness was achieved at 95% for the mentioned metal ions [62].

Despite the simplicity, high selectivity, and high removal efficiencies obtained in flotation, the process is constrained by the removal rate and ineffectiveness when applied to large dosage amounts of metals in water. In addition, if biosurfactants are not used, the process is constrained by high toxicity [62].

### 3.5. Electrochemical Separation

**Figure 2:**
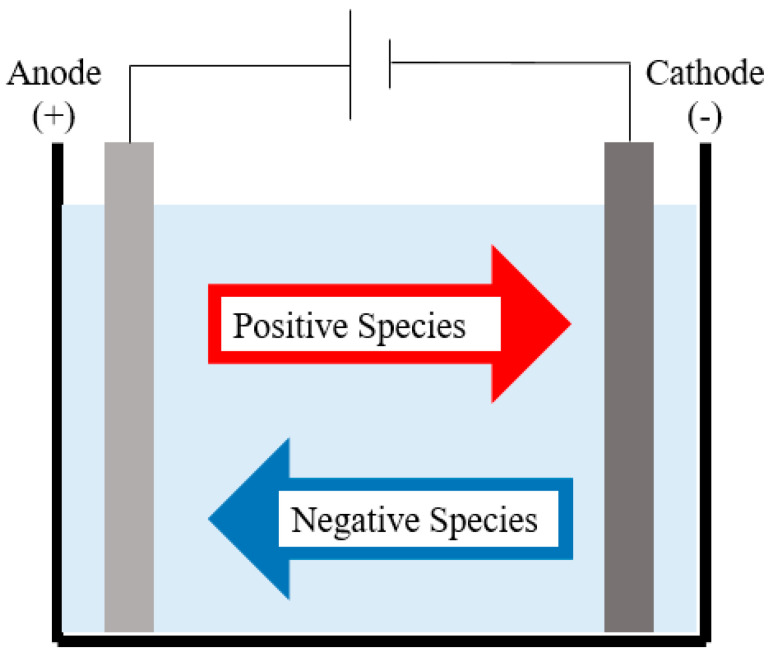
Electrochemical separation technique for wastewater treatment.

Electrochemical techniques have recently gained substantial attention and recognition as a dependable method for treating industrial wastewater. In contrast to alternative systems, electrochemical systems offer numerous advantages, such as operation under ambient pressure and temperature conditions, dependable performance, and the ability to accommodate fluctuations in influent flow rate and composition. This process is known for its ability to degrade various contaminants, including heavy metals. The fundamental aspect of the electrochemical reaction involves the flow of electric current through an aqueous solution containing metals, a cathode plate, and an insoluble anode (Figure 2) [63].

Heavy metals are managed through precipitation as hydroxides in electrolytes with a neutral or slightly acidic pH. In this way, the choice of electrode material plays a significant part in increasing the capabilities for various types of pollutants. Additionally, the number of ions created by the electrical current will determine the quality of the treated effluent. Therefore, any factor impacting the charge in the process will also affect the process’ overall efficiency [63].

Many metal ions, such as Hg^2+^, Cd^2+^, Pb^2+^, and Cu^2+^, were successfully removed with excellent efficiency using this process. To make this type of treatment viable for industrial applications, it is crucial to address the issue of energy consumption [64].

## 4. Elimination of Heavy Metals from Wastewater: Advanced Methods

Over time, the adoption of advanced technologies for removing heavy metals from wastewater has seen substantial growth. In the subsequent sections, we outline and explore some of the more prevalent advanced techniques, such as membrane filtration and adsorption, which include filtration systems. These methods exhibit numerous notable advantages in comparison to traditional approaches.

### 4.1. Membrane Filtration

Among the array of wastewater treatment methodologies, membrane filtration is frequently employed as a separation technique within water purification systems. Membrane filters can prevent the formation and spread of bacteria and viruses but also remove particles like total suspended solids, turbidity, and sediment from water.

**Figure 3:**
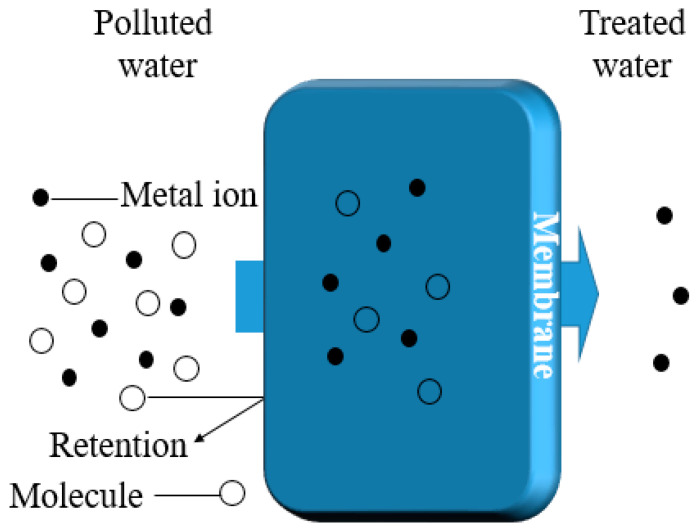
The generic membrane separation procedure.

**Figure 4:**
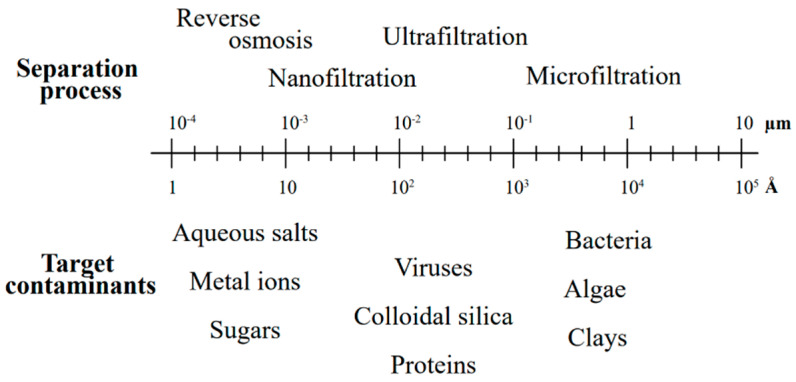
Filtration spectrum (source: adapted from [66]).

The attractive nature of membrane filtration to industry and central municipal wastewater recycling lies in its capability to remove suspended solids, organic compounds, and inorganic contaminants, including heavy metals. The pressure-driven membrane filtration process is primarily governed by the pressure difference between the feed and permeate sides (Figure 3) [65]. Various membrane filtration methods can be applied for the extraction of heavy metals from wastewater, depending on the desired particle size retention [15], as illustrated in the filtration spectrum adapted from [66] (Figure 4).

**Figure 5:**
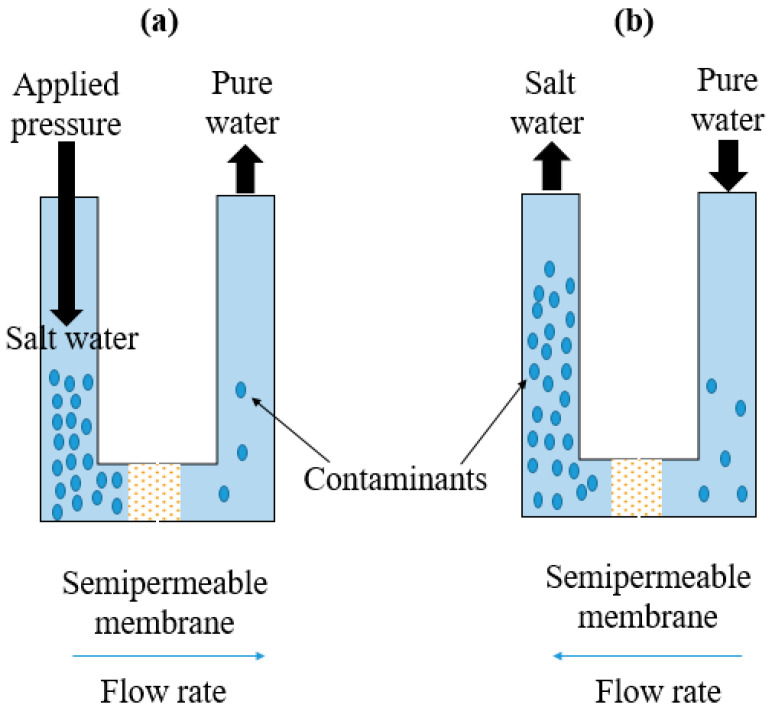
Reverse osmosis (**a**) and forward osmosis (**b**) membrane separation methodologies.

Reverse osmosis (RO) is a separation process driven by pressure, employing a semi-permeable membrane with a pore size of 0.5–1.5 nm. This membrane explicitly allows the passage of molecules smaller than the size of its pores (Figure 5a). The RO process works by exerting pressure values (ranging from 20 to 70 bar) higher than the feed solution’s osmotic pressure. This effectively reverses the typical osmotic process. The solute molecules within the solution generally have a molecular size ranging from 0.25 to 3 nm [67]. The filtration yield was estimated at 95–99% inorganic salts and charged organic matter removal. In the RO separation process, heavy metal ions present in electroplating wastewater, such as Ni^2+^, Cr^6+^, and Cu^2+^, are effectively removed with an efficiency surpassing 98.75% [68]. In recent times, the method has been utilized for the purification of industrial wastewater derived from mining activities in a coaster-field in Victoria-Australia, with averaged extraction efficiency values dependent on the extracted metal: Fe^3+^ (10%), Zn^2+^ (48%), As^3+^ (66%), Ni^2+^ (82%), and Sb^3+^ (95%) [44]. RO is a compact method with a high removal rate. Nonetheless, the exerted pressure can result in membrane fouling and deterioration as contaminants accumulate on the surface of the membrane [69].

Conversely, the forward osmosis (FO) procedure entails employing a semi-permeable membrane to partition the feed solution from the draw solution (Figure 5b). This approach balances selectivity and the flow rate of permeate water [70]. Typically, the draw solution has a higher osmotic pressure than the feed solution. This difference in osmotic pressure induces water movement from the feed solution to the draw solution, leaving behind undesired solutes in the feed solution while treating the water in the draw solution [71]. This method is eco-friendly and efficient, as it does not necessitate hydraulic pressure for the FO process. The devices based on FO are easy to clean, have a low fouling rate, and are widely used in wastewater treatment [63,64], with extraction efficiency reports for Pb, Cr, As, Cd, and Cu as high as 99.5%. In other studies, 98–99.96% removal efficiency was achieved in removing Co^2+^, Cu^2+^, and Zn^2+^ from acid mine drainage [72,73]. Despite its relatively effective removal capabilities, FO technology has its limitations. The choices for FO membranes are restricted [74], and the desired solute often needs to be regenerated through methods like membrane distillation [75,76], which can also entail increased energy consumption [72].

In contrast, in ultrafiltration (UF), both water and low-molecular-weight solutes permeate through the membrane, while larger macromolecules exceeding the membrane’s pore size are retained. UF typically possesses a pore size range of 0.001–0.1 μm, offering effective removal capabilities for wastewater treatment. Conversely, microfiltration (MF) normally operates with pore sizes that are up to an order of magnitude larger. While wastewater treatment often employs relatively open MF membranes with a pore size of 0.1–1.0 μm, the current trend in water treatment favors using moderately tight MF membranes with pore sizes ranging from approximately 0.09 to 0.1 µm [65]. The membrane filtration techniques applied to industrial wastewater can give effluent for disposal or feedstock for reusing [65]. UF membrane technology has been successfully used to eliminate heavy metals, such as iron, copper, and chromium, with a capacity ranging from 96 to 99% [77]. MF is usually used in municipal water treatment applications due to its removal capacity of viruses and, to some extent, its depth filter function. It also provides a barrier against bacterial and protozoan parasites [65].

Various other membrane types have been documented in the literature, including membranes composed of synthesized metal oxide/polyethersulfone (PES), Al_2_O_3_/PES, and ZrO_2_/PES. These membranes were employed for the removal of metals from wastewater. They were crafted by incorporating PES, Al_2_O_3_, and ZrO_2_ nanoparticles, with particle sizes ranging from 40 to 50 nm and surface areas measuring between 20 and 34 m^2^/g. The membrane fabrication process involved the utilization of the phase inversion method. To prepare the 5% w/w metal oxides/PES membranes, the metal oxide nanoparticles were dispersed in a solution of N-methyl pyrrolidone. The effectiveness of these membranes in removing Cr and Pb from both synthetic and natural wastewater was tested. The results showed that the membranes achieved approximately 99% removal for Pb and 88% for Cr [70].

**Figure 6:**
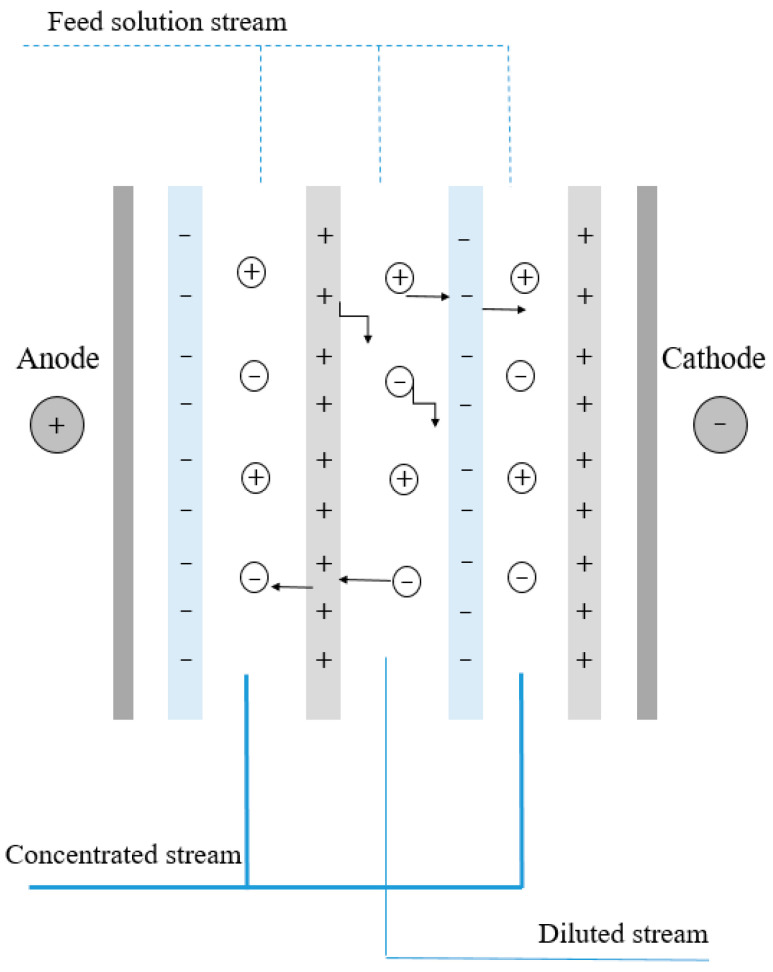
Electrodialysis membrane separation technique.

Electrodialysis (ED) is a membrane-based technology widely utilized for seawater desalination. It involves the arrangement of numerous anion exchange membranes (AEM) and cation exchange membranes (CEM) placed alternately between two electrodes (Figure 6). The electrolyte solution flows through the electrode compartment, while the feed solution traverses the adjacent compartment. When a voltage is applied, a reduction reaction occurs at the cathode, during which hydroxide ions are formed. Similarly, within the anode compartment, an oxidation process occurs, leading to the production of protons. There are slight variations in selectivity for different ions among various membranes used in ED, and the accumulation of divalent ions often leads to membrane fouling. Furthermore, these membranes tend to be expensive. Additionally, ion-exchange nanofibrous membranes are frequently regarded as a means to enhance the flow and permeability of membranes that selectively filter monovalent and divalent ions [78,79,80].

Furthermore, ED was used to separate Ni^2+^ and Pb^2+^ from synthetic solutions by a novel heterogeneous ED-CEM (consisting of 2-acrylamido-2-methylpropanesulfonic acid hydrogel and PVC) with extraction efficiencies of 96.9% and 99% for Ni^2+^and Pb^2+^, respectively. A pilot-scale ED system was employed to extract Cu^2+^, Ni^2+^, and small amounts of Cd^2+^, Fe^3+^, Cr^6+^, and Zn^2+^ with elimination efficiencies exceeding 90%. ED removes As^3+^ and As^5+^ in metallurgical wastewater, and the removal efficiency reached 91.38% [81].

Although membrane filtration has significantly progressed in laboratory-scale studies, no pilot-scale or large-scale practical applications have been reported. In addition, its main disadvantage is the sludge generation [15]. In contrast, membrane filtration operated through direct pressure can facilitate wastewater treatment with reduced energy consumption and carbon dioxide emissions. In summary, membrane filtration-based wastewater treatment systems hold the potential to attain net energy gains and additional economic benefits, paving the way for self-sustaining municipal wastewater treatment [65].

### 4.2. Adsorption

Adsorption is a good weapon in fighting against toxic metals threatening our environment. It is an interfacial physicochemical phenomenon accumulating solute molecules on a solid–liquid or solid–gas interface [82]. On the molecular scale, adsorption can be categorized into chemical and physical, contingent on the underlying interactions guiding the adsorption mechanism. The physical process assumes greater significance when it comes to the removal of heavy metals from wastewater.

**Figure 7:**
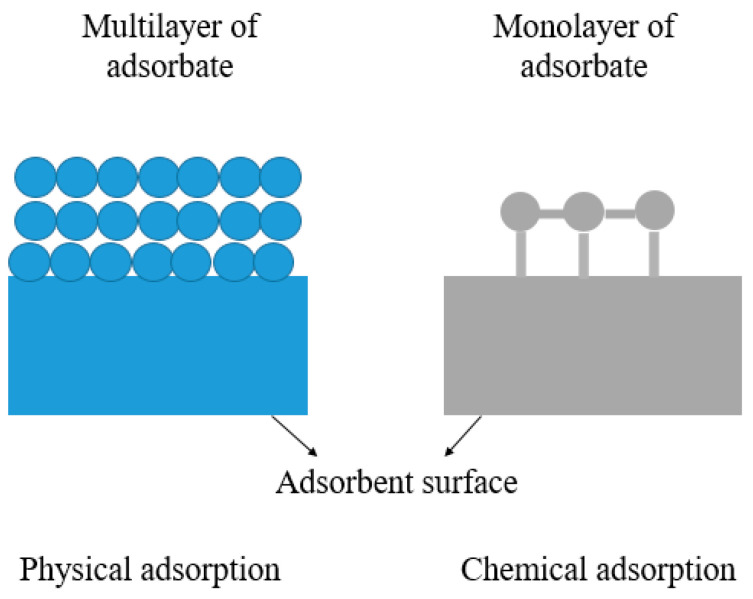
Physical and chemical adsorption.

In the case of physical adsorption, the Van der Waals molecular attractive forces that retain the adsorbent on the surface are generally dominant, and the phenomenon is reversible. The energy involved in the interaction between the adsorbate and the adsorbent shares a similar order of magnitude with the interaction energy between the two. Nevertheless, it typically exceeds the energy associated with the condensation of the adsorbate (Figure 7). Chemical adsorption, often referred to as activated adsorption, arises from a chemical bonding or interaction occurring between the solid substrate and the adsorbed substance.

The growing ability of adsorption to remove hazardous substances without compromising water quality or generating harmful degradation products has led to its increased utilization over electrochemical, biochemical, or photochemical degradation processes. Additionally, adsorption provides a valuable alternative treatment choice, particularly when the adsorbent is cost-effective and readily obtainable [83]. Various adsorbents have been explored and detailed in the existing literature, with these materials being obtainable from biological, organic, or mineral sources.

The significant costs tied to the creation and regeneration of activated carbon inspire researchers to explore alternative avenues. Furthermore, due to low metal adsorption capacity, synthetic materials like (E)-2-[(1H-imidazole-4-yl)methylidene]-Hydrazinecarbothioamide ligand (EIMH) often do not provide greater than 80% removal efficiency for heavy metals in wastewater treatment [84]. In recent years, novel adsorbent materials, particularly nanostructured substances like carbon nanotubes, graphene, fullerenes, and similar materials, have emerged. These materials are currently used to effectively adsorb 90% of heavy metals in wastewater because of their high specific surface area (500–1500 m^2^/g), large pore volume, and different intermolecular interactions [85]. Moreover, they exhibit non-corrosive properties and possess outstanding mechanical strength, thermal stability, and electrical conductivity. The adsorption performance of these nanostructured materials is generally better than that of classic synthetic adsorbents, such as iron oxide, titanium dioxide, activated carbon, etc. However, their limitations make scientific research challenging regarding functionality and durability, which are essential in environmental applications [86]. The adsorption efficiency can be improved by enhancing the carbon surface charge by adding surface functional groups such as carboxyl, phenyl, and lactone [87]. Surface modification typically reduces the surface area while increasing the concentration of surface functional groups. Conversely, increasing the surface area of the adsorbent, dosage of the adsorbent, initial concentration of metal ions, and contact duration tend to enhance adsorption. Multiwalled carbon nanotubes have received significant attention for effectively removing heavy metals [88]. Their high hydrophobicity and the presence of strong Van der Waals forces have generated considerable interest in their ability to swiftly accumulate in aqueous solutions, thus enhancing their adsorption capacity. The complex preparation process of carbon-based adsorbents contributes to their high cost and limits their widespread industrial use. Therefore, it is necessary to continue exploring innovative, affordable, and environmentally friendly surface modification technologies [88].

Chemical adsorbents encompass metal salt accelerators, activated silica, synthetic polymers, and natural accelerators. Among these, metal salt adsorbents are widely employed in sewage treatment plants due to their effectiveness in removing diverse pollutants like heavy metals and turbidity. The notable advantage of metal salt adsorbents over other types lies in their efficiency in tackling various pollutants. Metal salt adsorbents are readily accessible in the market and can deactivate bacteria. This affordability and availability make them popular among users, especially compared to other chemical adsorbents [89]. The metal salt adsorbents commonly utilized are those based on aluminum and iron [90,91]. They show good efficiency (90–99%) for removal of heavy metals like copper, lead, and nickel. In addition, they have the advantage of being less expensive and having a wider pH range. Adsorption has several disadvantages, including the high cost of adsorbent, separation of the adsorbent from metals, and low surface area [90,91].

#### 4.2.1. Filters: Classification and Applications

The treatment of industrial and domestic wastewater commonly utilizes adsorption-based filters due to their advantages over other methods, such as cost reduction and lower energy consumption. However, several factors need to be considered and optimized for the optimal performance of these filters. The primary factors include the choice of adsorbent material, which significantly impacts heavy metal removal efficiency. Adsorbent properties such as specific surface area, pore size distribution, and functional groups play a crucial role [92]. Additionally, the characteristics of the heavy metals themselves, including their concentration and the presence of competing ions, must be taken into account [93]. Operating conditions, such as pH, temperature, and contact time, are also critical to adsorption [94]. Furthermore, the design and configuration of the filters, encompassing parameters like porosity, depth, flow rate, and material leakage into the water effluents, require careful consideration and improvement [95]. Specific factors such as the regeneration and reusability of the adsorbents [96], the particle size distribution [97], and the potential for chemical modifications to enhance adsorption capacity are equally important [98]. The subsequent sections of this review delineate three categories of filters: synthetic, natural, and hybrid, each with unique properties and applications.

##### Synthetic Filters

**Figure 8:**
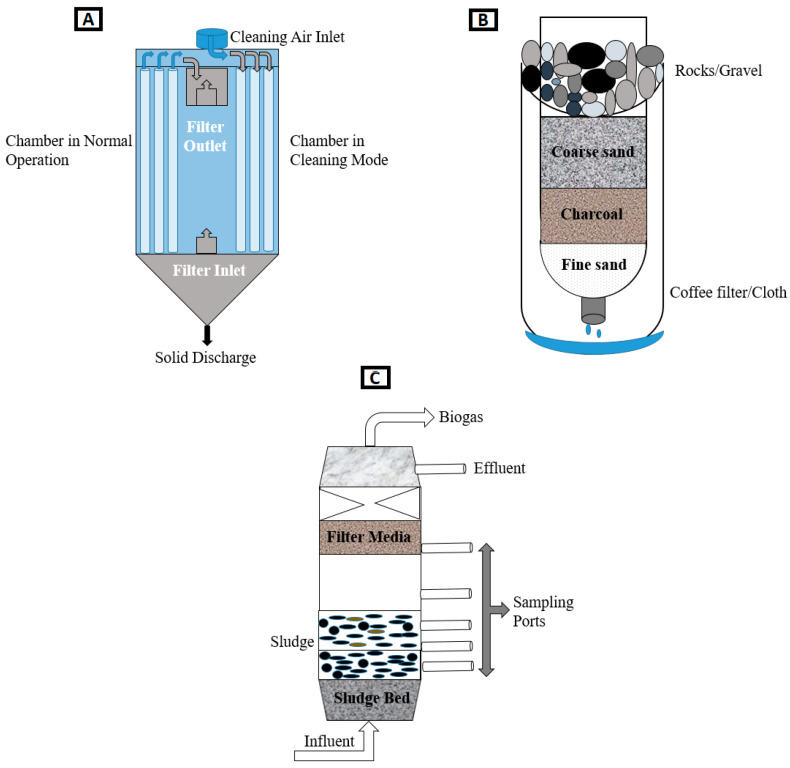
(**A**) Synthetic filter, (**B**) natural filter, and (**C**) hybrid filter.

Among the many synthetic filters designed and studied in the literature, polymer-enhanced filters perform relatively well in removing heavy metals from water. They consist of deploying water-soluble polymeric ligands able to bind metal ions selectively, producing metals-free effluents. Figure 8A presents a schematic depiction of a synthetic filter’s distinct constituents. To illustrate the different metals that such filters can remove and their filtration efficiency, Table 2 summarizes various important examples of synthetic filters described in the literature.

A filter layer may comprise a semi-interpenetrating polymer network composed of cross-linked polyvinyl alcohol (PVA) as the matrix, with the effective utilization of PEI for the removal of heavy metal ions, including Pb^2+^, Cd^2+^, and Cu^2+^ from aqueous solutions. The PVA chains of the complexing polymer are entrapped in a cross-linked polymeric matrix. The latter is fabricated through exposure to gaseous 1,2-dibromoethane. The adsorption reactions followed pseudo-first-order kinetics with similar rate constants for the three cations, and the obtained equilibrium constants show the following affinity order: Pb > Cu > Cd [99].

In addition, PAN demonstrates a remarkable ability to effectively adsorb diverse metal ions, rendering it highly appealing for purification purposes. Notably, it is considered one of the most cost-effective polymers due to its abundant cyano groups at the ends, which provide ample opportunity for further modification [105,106]. PAN fibers are produced through free radical bulk polymerization, utilizing benzoyl peroxide as an initiator. To achieve a bead-free and consistent morphology, a series of experiments were carried out at different potential differences and flow rates. The electrospinning process was optimized at a potential difference of 3 kV cm^−1^ and a flow rate of 2 mL h^−1^. At room temperature, the nitrile groups on the PAN fibers’ surface reacted with H_2_NOH. Hydroxylamine engaged in a nucleophilic addition to the fiber surface, subsequently forming carboximides. The fiber surface was then functionalized to chelate uranyl cations, and the resulting fiber could remove uranyl ion U(VI) pollutants [107]. By comparing the different PAN fibers, good sorption results of U(VI) ions were obtained (81%). In contrast, only 19% sorption was obtained when the PAN fibers remained unmodified. Most importantly, nanofibers have a high surface area, enhancing their adsorption efficiency and making them ready to link to various chelating agents [108].

Recently, a new filter to remove Cd^2+^ and Pb^2+^ from the water was described in the literature. It consisted of fly ash ceramic foams with a 3-D interconnected porous structure and multiwalled carbon nanotubes (MWCNTs) that are used by a combination of carbamate grafting and polydimethylsiloxane coating. The fly ash foam exhibited removal rates of 96.33% and 95.12% for Cd and Pb, respectively. This synthetic filter can save considerable economic effort due to gravity propulsion. It is suitable for front-end wastewater treatment on an industrial scale [104].

In a different investigation, PVA/SA beads were formulated by blending functionalized sodium alginate (SA) with polyvinyl alcohol (PVA), including glutaraldehyde as a cross-linking agent. Zeo/PVA/SA nanocomposite beads were made using zeolite nanoparticles (Zeo) and PVA/SA to remove heavy metals from wastewater with good efficiency: 99.5% (Pb^2+^), 99.2% (Cd^2+^), 98.8% (Sr^2+^), 97.2% (Cu^2+^), 95.6% (Zn^2+^), 93.1% (Ni^2+^), 92.4% (Mn^2+^), and 74.5% (Li^2+^) [109].

Using synthetic adsorbents in filters presents an opportunity to explore the binding mechanisms of heavy metals with different compounds, whether in a specific or non-specific manner. The polymer-based adsorbents are particularly easy to produce and have been extensively used. The manufacture of these synthetic filters demands a substantial amount of energy. Furthermore, they are not environmentally friendly, as they are typically discarded after becoming saturated with pollutants, unlike natural or hybrid filters, which can be reused.

Furthermore, the production of synthetic zeolites involves using numerous chemical materials. While industrial waste products like cupola slag and spent fluid cracking catalysts are employed in synthesizing important zeolites (such as ZSM-5, NaA, and NaX), they pose environmental hazards. Additionally, specific types of municipal solid waste, such as non-recyclable glass and thin-walled aluminum scraps (including aluminum cans and foils), can serve as sources of silicon (Si) and aluminum (Al) for zeolite synthesis. In summary, zeolite stands as an exceptional adsorbent for effectively removing heavy metals from wastewater [110].

##### Natural Filters

Natural wastewater treatment systems, including constructed wetlands, biological sand filters, and other decentralized solutions, are gaining significance as viable alternatives to previously discussed systems (Figure 8B). This is primarily due to their low establishment costs, ease of use, minimal management requirements, and high efficiency in removing heavy metals from water [111,112].

In less-developed countries, natural coagulants are commonly employed as point-of-use technology due to their ability to be locally produced and easily processed into a usable form. These coagulants are biodegradable and present a cost-efficient substitute for chemical coagulants. Their mode of action involves an adsorption mechanism, followed by either charge neutralization or a polymeric combining effect [113]. Starch and cellulose derivatives, proteinaceous materials, and gums from polysaccharides are significant natural products used [88]. For example, Moringa and other native seeds can remove metals from drinking water. Table 3 shows that Moringa seeds have higher removal rates of heavy metals than cowpea (*Vigna unguiculata*), urad (*Vigna mungo*), peanuts (*Arachis hypogaea*), and corn (*Zea mays*). Moringa has a metal removal rate of over 50%, with the highest removal efficiency for copper and lead (90% for both). Beans (black-eyed peas) and peanuts do not have high metal removal efficiency (<15% for Cu and Cd). Corn and urad beans exhibit limited capabilities for the removal of heavy metals. The adsorption of metals using Moringa is restricted to its surface, as Moringa is a cationic polyelectrolyte with a short chain and low molecular weight [114]. Compared to other seeds, Moringa is particularly effective in efficiently removing heavy metals and solids with charges higher than its colloidal surface. Nonetheless, additional research is necessary to gain a deeper understanding of the molecular mechanisms underpinning this effective water purification. This insight will be crucial in refining the design of filters employing Moringa.

Large amounts of biomass waste, peels, leaves, husks, stems, branches, and pods can be valorized and used in biosorption [125]. Therefore, natural filters using biomass waste adsorbents are green and sustainable filtration devices. For example, natural zeolites have high ion exchange and adsorption performance due to their particular structure. The ion exchange capacity is influenced by various factors, such as the structure of the framework, the size and shape of the ions, the charge density of the anionic framework, the ionic charge, and the concentration of the external electrolyte solution [126,127].

Given the numerous advantages, the development and utilization of natural coagulants have promising prospects as an environmentally friendly and sustainable technology for wastewater treatment [128]. Without a doubt, the drawbacks of employing natural adsorbents primarily stem from the characteristics of the adsorbents themselves. Factors like seasonal variations and storage duration can impact natural adsorbents’ production and consistent availability [129,130,131].

##### Hybrid Filters

Although natural filters are environmentally sustainable and broadly applied in water filtration, their affinity for heavy metals remains moderate, and this finding makes the raw materials less attractive. Improved heavy metal retention from wastewater has been obtained for filters that include modified natural products. Such filtration devices are called hybrid filters. Given their setup composition, they are environmentally sustainable. Figure 8C shows a schematic representation of a hybrid filter.

Two exemplary hybrid filters will be discussed in this context, specifically those based on beetroot and limestone. Beetroot fibers, subjected to chemical modification using sodium dodecyl sulfate (SDS), an anionic surfactant, were prepared as representative examples. They proved their efficiency in cleaning water contaminated by organic and inorganic compounds and softening hard water (Table 4). In this specific system, Rahman et al. pinpointed the factors influencing wastewater purification efficacy, which include fiber particle size, initial pollutant concentration, and wastewater pH. The findings indicate that the purification efficiency increases as the fiber size decreases (from millimeters to micrometers). The optimal pH for adsorption was determined to be in the range of 6.0–6.5. Next, the modified filter’s dual function was investigated by assessing its ability to remove heavy metals and soften hard water through its chemical functionalities’ positive and negative charges. Hybrid filters based on beetroot fibers were able to remove various pollutants: metals (lead, zinc, nickel, copper), total solid dissolved, and methylene blue. They exhibited high efficiency when particle size decreased due to increased contact surface area at pH values from 6.0 to 6.5. The maximum removal was 70 mg/g of filter for heavy metals like Pb, Cu, and Zn [132].

The second hybrid filter example is based on limestone, a material with sedimentary rock origin consisting mainly of CaCO_3_, which, in turn, comes from calcite and then aragonite. The chemical composition of limestone renders it an appropriate material for water and wastewater treatment, as these elements possess the capability to adsorb a diverse range of pollutants. While limestone is an eco-friendly substance extensively utilized in water filtration, it exhibits a moderate affinity for heavy metals. Consequently, endeavors have been dedicated to developing an environmentally friendly filtration system by combining limestone and activated carbon to eliminate iron from wastewater. A filter composed of activated carbon and limestone in a 1:1 ratio successfully removed a 10 mg/L iron solution. Utilizing 1 kg of this filter, it only took 95 min, at pH 6, to completely eliminate iron ions from the contaminated solution [136].

Furthermore, hybrid inorganic/natural adsorbents primarily based on alumina and 4-amino antipyrine have been implemented to remove Pb, Cu, and Cr from industrial wastewater. The hybrid adsorbents have been effectively implemented for the selective elimination of the heavy metals mentioned above, with recuperation values between 92 and 98%. Hybrid alumina adsorbents are known for their exceptional thermal decomposition resistance and a sturdy balance that approaches acid-leaching conditions [141]. They were recently replaced by natural adsorbents, representing a safe, more eco-friendly, and energetically cheaper alternative [142]

Furthermore, modified zeolite can be easily obtained through basic or acidic treatment and surfactant modification. These alterations expand the range of potential applications for zeolites in wastewater treatment. As a result, surfactant-modified zeolites have the capability to not only adsorb anionic species but also undergo cation exchange with metal cations. This is due to the large size of the modifying agents’ cations, preventing them from entering the inner channels [110].

#### 4.2.2. Adsorbents Regeneration/Reuse

Spent adsorbents require regeneration after reaching their saturation point with contaminants to restore their adsorption capacity and facilitate reuse. The reusability of spent adsorbents depends on their recoverability, decontamination efficacy, and regeneration potential [143]. High recovery and regeneration capabilities are highly desirable for commercial and industrial applications, as they can significantly reduce the overall cost associated with adsorbent production [144]. Although the regeneration process can be cycled multiple times, regenerated adsorbents typically show a decrease in adsorption capacity compared to fresh ones [145]. Selecting the most appropriate regeneration technique is crucial for maximizing contaminant desorption efficiency. Several factors influence the feasibility of industrial-scale application, including the type of adsorbent, contaminant, adsorbent stability, toxicity, and the cost and energy demands of the regeneration process.

A diverse range of approaches can be employed to recover and regenerate spent adsorbents. These include magnetic separation [146], thermal desorption [147], solvent regeneration [148], microwave irradiation [149], supercritical fluid regeneration [150], and advanced oxidation processes [151].

- Magnetic separation leverages the magnetic properties of adsorbents to separate them from the wastewater after they have adsorbed heavy metals. By applying an external magnetic field, the magnetic adsorbents can be easily retrieved from the solution, facilitating their regeneration and reuse. The primary advantages are efficiency and simplicity, allowing for quick recovery of adsorbents, reducing the need for complex filtration methods, and lowering operational costs while minimizing secondary pollution [152,153]. However, the stability of the magnetic properties over multiple regeneration cycles needs to be ensured.
- Thermal desorption involves heating the adsorbent to volatilize the adsorbed metals, which are then captured and treated separately. Regeneration temperatures typically range from 200 to 800 °C [147]. Thermal desorption can achieve high regeneration efficiencies, often exceeding 90%, making it suitable for large-scale industrial applications [152]. It is effective for various adsorbents, including activated carbons, zeolites, and metal-organic frameworks (MOFs), but has drawbacks like high energy requirements and potential degradation of adsorbents over multiple cycles.
- Solvent regeneration involves using various solvents to desorb metals from the adsorbent surface, effectively restoring its adsorption capacity. Common solvents include acids (e.g., hydrochloric acid, nitric acid), alkalis (e.g., sodium hydroxide), and organic solvents (e.g., ethanol, methanol) [148,152,154]. This method is particularly effective for adsorbents with a strong affinity for certain heavy metals and is relatively cost-effective. However, it poses environmental risks due to the disposal of spent solvents and can lead to partial degradation of the adsorbent material over time. Recent advancements focus on developing eco-friendly solvents and integrating solvent recovery systems to enhance sustainability [152,154].
- Microwave irradiation heats the adsorbent using microwave energy, volatilizing the adsorbed contaminants and allowing for efficient desorption and regeneration. Studies have shown that microwave-assisted regeneration can recover up to 90% of the adsorptive capacity of activated carbon used in wastewater treatment [152,155]. This method is highly effective at targeting specific adsorbed species and is energy-efficient and environmentally friendly, offering rapid and selective heating that reduces operational costs and environmental impact [149].
- Supercritical fluid regeneration uses supercritical fluids, typically supercritical CO_2_, which operate under conditions above their critical temperature and pressure. This method achieves high efficiency in desorbing heavy metals from adsorbents and can achieve metal recovery rates exceeding 90%, making it highly effective for industrial applications. Supercritical CO_2_ is non-toxic, non-flammable, and relatively low-cost. The process minimizes waste and environmental impact and preserves the structural integrity of the adsorbent material over multiple regeneration cycles. However, it requires high initial setup costs and precise control of operational parameters to optimize regeneration efficiency [156].
- Advanced oxidation processes (AOPs) generate highly reactive species, such as hydroxyl radicals, which degrade contaminants adsorbed onto the adsorbents, restoring their adsorption capacity [157]. AOPs can regenerate adsorbents without significant material degradation, allowing for multiple reuse cycles and reducing operational costs. These processes target a wide range of heavy metals, ensuring comprehensive removal and recovery. However, they require high initial setup costs and precise control of operational parameters [157].

## 5. Comparison between Filters and Other Methods

**Figure 9:**
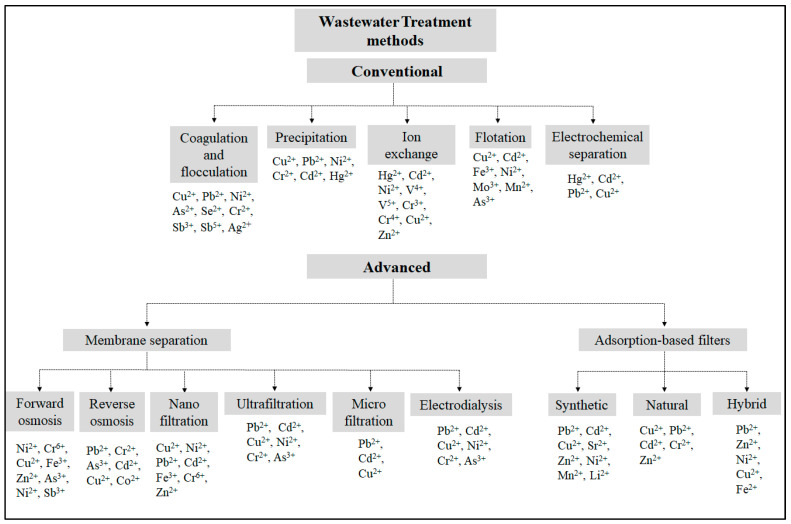
Summary of the conventional and advanced wastewater treatment methods described in this review.

The need for cost-effective technologies to remove heavy metals from contaminated soil and water is rising. The conventional and advanced metal removal methods described in this review are summarized in Figure 9. The conventional methods have become insufficient to meet the current strict regulatory effluent restrictions and are sometimes expensive [128]. Adsorption is a commonly employed method for extracting toxic metals from aquatic environments, achieving an average removal rate of around 99% through the use of inexpensive adsorbents, including agricultural waste and activated carbon derived from agricultural waste [158,159]. Therefore, there is a great demand for alternative, cost-effective technologies. Selecting a specific treatment method relies on various factors, including the waste’s type and concentration, the effluent’s variability, the desired remediation level, and economic and energy considerations [40]. For example, filters use less energy than osmosis pumps, which remains true when compared to pressurized nano and ultra-filtration phenomena [68]. The pros and cons of different techniques for addressing heavy metals in wastewater are outlined in Table 5.

## 6. Conclusions and Perspectives

Ensuring the well-being of both human health and the environment entails the vital mission of eradicating heavy metals from wastewater. A plethora of treatment approaches, encompassing adsorption, membrane filtration, coagulation and flocculation, chemical precipitation, ion exchange, flotation, electrochemical processes, and filtration, have been the subject of extensive research and have proven their effectiveness in the removal of heavy metals from wastewater. Each method has advantages and limitations, and further research and development are needed to optimize efficiency, cost-effectiveness, and environmental sustainability. The integration of multiple treatment methods and the development of hybrid systems can enhance the overall effectiveness of heavy metal removal. Additionally, using advanced materials, such as nanostructured materials, natural coagulants, and modified natural products, shows promise in improving heavy metal removal efficiency.

Natural filters are cheaper and more eco-friendly than hybrid filters but are less efficient than synthetic ones in removing organic and inorganic pollutants. To limit the impact of such filters on the environment, efforts should be invested to develop efficient biomass-based biodegradable filters for water treatment. A sustainable and economically viable strategy for designing such filters is the use of what is currently considered as biomass-waste. Coupling between various sorts of natural and hybrid filter treatment could also be a promising alternative. Furthermore, adopting sustainable wastewater treatment practices and promoting public awareness and education are essential in addressing global water pollution. By continuing to advance and implement efficient and sustainable wastewater treatment technologies, we can mitigate the harmful effects of heavy metals, protect water resources, and ensure a cleaner and safer environment for future generations.

## Figures and Tables

**Table 2:** Metal ions elimination by synthetic filters.

Synthetic Filter	Targeted Metals	Metal Ions Removal (%)	Reference
PEI	Pb^2+^	80	[99]
	Cu^2+^	94	
	Cd^2+^	99	
PAN	Cu^2+^	96	[100]
Synthetic Zeolites	Pb^2+^	95	[101]
	Cd^2+^	90	
	Ni^2+^	85	
Dithiocarbamate resins	Pb^2+^	45	[102]
	Cu^2+^	78	
	Hg^2+^	80	
	As^3+^	55	
	Cr^3+^	76	
Metal Oxides (e.g., MnO₂, Fe₃O₄)	As^3+^	80	[103]
	Pb^2+^	85	
	Cd^2+^	65	
	Cr^3+^	55	
MWCNTs	Cd^2+^	96	[104]

**Table 3:** Removal capacities of natural adsorbents.

Natural Adsorbents	Removal Capacity (%)					Reference
	Cu^2+^	Pb^2+^	Cd^2+^	Cr^3+^	Zn^2+^	
Urad	30	40	10	20	15	[115]
Peanut	4	50	8	40	22	[116]
Bean	3	29	10	40	30	[117]
Corn	10	13	40	10	20	[118]
Moringa	90	90	60	50	55	[119]
Clay Minerals (e.g., Bentonite, Kaolinite)	-	80	70	50	-	[120]
Zeolites	90	75	-	-	60	[121]
Peat Moss	52	50	70	-	-	[122]
Sawdust	60	20	30	80	-	[123]
Algae (Biomass)	50	40	-	60	45	[124]

**Table 4:** Metal ions elimination by a hybrid filter.

Hybrid Filter	Targeted Metals	Metal Ions Removal (%)	Reference
Beetroot fibers and SDS	Pb^2+^	100	[132,133,134,135]
	Zn^2+^	99	
	Ni^2+^	99	
	Cu^2+^	99	
Limestone and activated carbon	Fe^2+^	100	[136,137,138]
Clay-EDTA	Pb^2+^	95	[139]
Chitosan Modified with Thiol Groups	Hg^2+^	90	[140]

**Table 5:** The primary benefits and drawbacks of diverse traditional and advanced approaches, including filtration methods, for eliminating heavy metals from wastewater.

Treatment Method		Advantages	Disadvantages	Reference
Coagulation and flocculation or precipitation		Low cost; simple operation	Sludge generation; the extra operational cost of sludge disposal	[160]
Ion exchange		Removal of metals and organic pollutants simultaneously; less harmful byproduct	Long duration time; limited application	[161]
Membrane filtration, forward and reverses osmosis		Small space requirement; low pressure; high separation selectivity	High operational cost due to membrane fouling	[160]
Adsorption		Availability; high efficiency; less expensive than other techniques; works in a wide pH range	Hard separation of the adsorbent from metals	[162,163]
Adsorption by Filters	Synthetic filters	High efficiency	High energy consumption	[164]
	Natural filters	Low-cost; eco-friendly	Moderate efficiency	[165]
	Hybrid filters	High separation selectivity; economical; 50% eco friendly	50% harmful effect on the environment	[166]

## Data Availability

The data presented in this study are available in the article.
